# Severe Lymphocytosis in a Case of Diffuse Large B-Cell Lymphoma Treated by Ibrutinib

**DOI:** 10.4274/tjh.galenos.2021.2021.0362

**Published:** 2021-12-07

**Authors:** Semra Paydaş, Ertuğrul Bayram, Mehmet Türker, Turan Özer

**Affiliations:** 1 Çukurova University Faculty of Medicine, Department of Medical Oncology, Adana, Turkey

**Keywords:** Ibrutinib, Lymphocytosis, Diffuse large B cell lymphoma

## To the Editor,

We would like to report extreme leukocytosis in a case of diffuse large B-cell lymphoma (DLBCL) treated by ibrutinib [1]. To the best of our knowledge, this is the first case of a very high white blood cell (WBC) count in a patient with DLBCL.

A 63-year-old man was diagnosed with stage IV-B DLBCL with bone marrow (BM) infiltration. He had double-hit lymphoma and Ki67 was 90%. Two cycles of a dose-adjusted rituximab, etoposide, prednisone, vincristine, cyclophosphamide, and doxorubicin (R-EPOCH) regimen were given with clinical and radiologic improvement. However, lymphomatous skin lesions confirmed by cytologic examination developed. Computerized tomography (CT) scans showed progressive disease. In the original biopsy sample, PD-L1 expression was detected in 30% of lymphoma cells. A rituximab-vinorelbine-gemcitabine-prednisolone regimen was given for two cycles as second-line treatment. However, rapid progression developed and an ibrutinib-nivolumab combination was planned and prescribed. Four days after ibrutinib treatment, WBC and lymphocyte/monocyte counts increased rapidly and peaked at 100x10^9^/L on the 20^th^ day of treatment. Mononuclear cells were larger than mature lymphocytes with nucleolus-like bodies. Flow cytometric surface analysis showed CD10: 96%, HLA-DR: 55.8%, cCD79a: 53.6%, CD45: 100%, CD19: 13%, CD20: 0% expression. Nivolumab was given on the 14^th^ day of ibrutinib treatment. CT scans showed regression of abdominal lymph nodes. However, febrile complication developed and he died due to infection.

It is well known that lymphocytes with CD19 and CD5 positivity and CD3 negativity increase in such cases [2]. Lymphocytosis in cases of chronic lymphocytic leukemia (CLL) is due to tumor cells moving from infiltrated tissues to the blood. This phenomenon is associated with a class effect and driven by the efflux of cells from tissue compartments, paralleled by a substantial decrease in total tumor burden [2,3,4].

**Figure 1:**
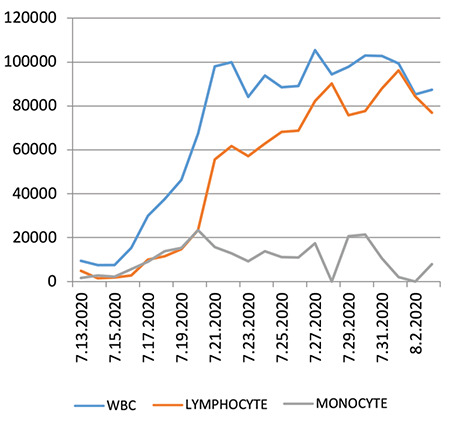
White blood cell (WBC), lymphocyte, and monocyte counts during ibrutinib treatment (/mm^3^).

Lymphocytosis has been reported in 34% of cases of mantle cell lymphoma (MCL) [5]. Furtado et al. [6] reported lymphocytosis in cases of MCL treated by ibrutinib among patients with higher BM infiltration. The efficacy and safety of ibrutinib in cases of DLBCL have been analyzed in a meta-analysis; the overall response rate was found to be 57.9% and 49.7% in cases of newly diagnosed DLBCL and relapsed/refractory cases of DLBCL, respectively [7]. We did not find any reports about lymphocytosis in cases of DLBCL treated by ibrutinib. Our patient had 100% BM infiltration and mononuclear cells increased rapidly with a peak at the 20^th^ day of ibrutinib treatment (Figure 1). The very high volume of mononuclear cells was due to the BM infiltration and also the presence of very high tumor burden. The CD20 negativity in peripheral blood may be due to the previous rituximab treatment. To the best of our knowledge, this is the first case of a very high lymphocyte count in a patient with DLBCL treated by ibrutinib. The mechanism of this lymphocytosis is associated with mobilization of malignant cells related to the action of lymphocyte trafficking, and egress of lymphocytes from the protective stromal microenvironment occurred in our case as also seen in cases of CLL treated by Bruton’s tyrosine kinase [8].

We suggest that peripheral blood cell counts may increase in patients with DLBCL with BM infiltration and/or high tumor burden as seen in CLL.
